# Fullerene C_60_ Conjugate with Folic Acid and Polyvinylpyrrolidone for Targeted Delivery to Tumor Cells

**DOI:** 10.3390/ijms25105350

**Published:** 2024-05-14

**Authors:** Alina A. Borisenkova, Olga I. Bolshakova, Anna V. Titova, Irina S. Ryabokon, Maria A. Markova, Zhanna B. Lyutova, Victor P. Sedov, Elena Yu. Varfolomeeva, Vadim V. Bakhmetyev, Alexandr V. Arutyunyan, Vladimir S. Burdakov, Svetlana V. Sarantseva

**Affiliations:** 1 Radiation Technology Department, St. Petersburg State Institute of Technology (Technical University), 190013 St. Petersburg, Russia; 2 Petersburg Nuclear Physics Institute Named by B.P. Konstantinov of National Research Centre “Kurchatov Institute”, 188300 Gatchina, Russia

**Keywords:** targeted drug delivery, fullerene, folic acid, polyvinylpyrrolidone, folate receptor, aggregation, cytotoxicity, internalization, radical scavenging activity

## Abstract

The use of targeted drug delivery systems, including those based on selective absorption by certain receptors on the surface of the target cell, can lead to a decrease in the minimum effective dose and the accompanying toxicity of the drug, as well as an increase in therapeutic efficacy. A fullerene C_60_ conjugate (FA-PVP-C_60_) with polyvinylpyrrolidone (PVP) as a biocompatible spacer and folic acid (FA) as a targeting ligand for tumor cells with increased expression of folate receptors (FR) was obtained. Using ^13^C NMR spectroscopy, FT-IR, UV-Vis spectrometry, fluorometry and thermal analysis, the formation of the conjugate was confirmed and the nature of the binding of its components was established. The average particle sizes of the conjugate in aqueous solutions and cell culture medium were determined using dynamic light scattering (DLS) and nanoparticle tracking analysis (NTA). The FA-PVP-C_60_ showed antiradical activity against ^•^DPPH, ^•^OH and O_2_^•**−**^, but at the same time, it was shown to generate ^1^O_2_. It was found that the conjugate in the studied concentration range (up to 200 μg/mL) is non-toxic in vitro and does not affect the cell cycle. To confirm the ability of the conjugate to selectively accumulate through folate-mediated endocytosis, its uptake into cells was analyzed by flow cytometry and confocal microscopy. It was shown that the conjugate is less absorbed by A549 cells with low FR expression than by HeLa, which has a high level of expression of this receptor.

## 1. Introduction

One of the disadvantages of standard chemotherapy approaches to treating cancer is the inappropriate distribution of drugs in the body. In the case of brain tumors, the therapeutic challenge is compounded by the blood–brain barrier. Therefore, it is extremely important to develop delivery systems directed to specific tumor cells, including receptor-mediated pathways [1]. It is known that in some types of tumors there is an increased expression of folate receptors (FR) [2,3], responsible for the delivery of 5-methyltetrahydrofolate, which is the cofactor necessary for cell proliferation. Since folic acid (FA) conjugates can bind to FR on the cell surface with properties similar to free folate [4,5], biocompatible conjugates of FA with antibodies, anticancer drugs, fluorescent labels, contrast and other agents are currently being widely investigated [6,7,8,9,10], while others are undergoing preclinical and clinical trials [3]. Conjugates of FA with carbon nanoparticles [11] and, in particular, with fullerenes [12,13] may become promising drugs for targeted delivery to tumor cells, including those resistant to standard chemotherapy [14]. The unique physicochemical properties of fullerenes, as well as their ability to penetrate the cell membrane [15,16], suggest their applicability in biomedicine, in particular, as vectors for drug delivery [14,17]. A number of studies have demonstrated that fullerenes and their derivatives have antitumor activity [18,19,20]. In addition, the use of fullerenes in photodynamic therapy [21,22,23] and as carriers of radioactive atoms for the treatment or diagnosis of oncological diseases [24,25,26] seems very interesting. Based on endohedral metallofullerenes, a new class of drugs for MRI with improved properties can be developed [27,28]. Moreover, their use will allow reducing the toxicity of metals used for these purposes. However, non-functionalized fullerenes are insoluble in water and have low biocompatibility. The most common solution to this problem is the synthesis of hydroxyl derivatives of fullerenes, namely fullerenols [29,30,31] and complexes of fullerenes with biocompatible polymers, such as polyvinylpyrrolidone (PVP) [32,33], polyethylene glycol [34,35], γ-cyclodextrin [36,37], dextran [38] and others. At the same time, the synthesis of such water-soluble fullerene derivatives does not solve the problem of their selective accumulation in tumor cells, whereas the creation of conjugates, including with FA, allows us to overcome this problem [39].

The aim of the present work is to obtain a C_60_ fullerene conjugate with FA as a targeted fragment for folate receptors, also containing PVP as a biocompatible hydrophilic spacer, which improves the bioavailability of poorly soluble drugs in water and is used as a carrier in controlled release systems [40]. The aggregation of the FA-PVP-C_60_ conjugate, its anti- and prooxidant activity and its toxicity towards normal and tumor cells were assessed, and the analysis of its internalization into cells with different levels of FR expression was conducted.

## 2. Results and Discussion

### 2.1. Characterization of the FA-PVP-C_60_ Conjugate

The advantage of polymer/fullerene complexes is considered to lie in the weaker perturbation of the fullerene’s π-electron system by the polymer compared to the change that occurs during the formation of covalent bonds between them. Standard methods for the synthesis of polymer/fullerene donor–acceptor complexes significantly limit the fullerene content in the complex, not exceeding 2 wt.% [41]. Obtaining complexes with a high C_60_ content is associated with significant difficulties caused by the lack of a common solvent for the components. In particular, this process is complicated by the aggregation of fullerenes in solvents [42]. At the same time, we needed to conjugate C_60_/PVP and FA into a complex. Therefore, for the synthesis of FA-PVP-C_60_, a one-component polar aprotic solvent, N,N-Dimethylformamide (DMF), was chosen, which dissolves PVP well and forms fairly stable colloidal solutions of C_60_ [43], which allowed obtaining conjugates with a fullerene content of up to 10 wt.%. However, with such a C_60_ content in the conjugate, its solubility in water did not exceed ~10 mg/mL. Therefore, we selected the optimal fullerene content in the conjugate of 2.8 wt.%, at which the solubility of the conjugate in water is quite high and amounts to ~50 mg/mL, ensuring good bioavailability of the resulting compound.

**Figure 1:**
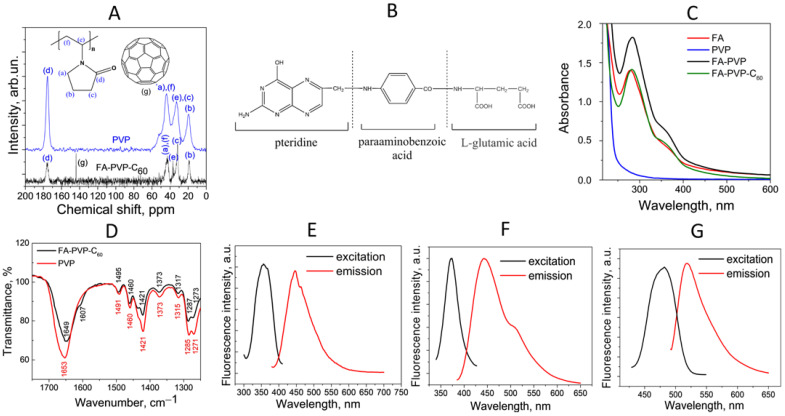
FA-PVP-C_60_ conjugate characterization. (**A**) Solid-state ^13^C-NMR spectra of FA-PVP-C_60_ (black line) and pure PVP (blue line). (**B**) The structure and characteristic parts of the FA molecule. (**C**) UV-Vis spectra of FA-PVP-C_60_, intermediate conjugate FA-PVP, and pure FA and PVP in water. (**D**) FT-IR spectra of FA-PVP-C_60_ conjugate and pure PVP. (**E**–**G**) Fluorescence spectra of FITC-labeled FA-PVP-C_60_ conjugate for various fluorescence excitation wavelengths.

The purity of fullerene C_60_, which was one of the starting reagents for the synthesis of the conjugate, was confirmed using HPLC (Figure S1A, Supplementary Materials), UV-Vis spectroscopy (Figure S1B, Supplementary Materials) and FT-IR spectrometry (Figure S1C, Supplementary Materials). The confirmation of C_60_ present in the conjugate was performed by solid-state ^13^C NMR spectroscopy (Figure 1A) and UV-Vis spectroscopy of FA-PVP-C_60_ in DMF (Figure S2B, Supplementary Materials). In the ^13^C NMR spectrum of FA-PVP-C_60_ (Figure 1A), the peak is around 140 ppm, corresponding to fullerene C_60_, and the other peaks correspond to pure PVP [44]. The UV-Vis spectrum of FA-PVP-C_60_ in DMF (Figure S2B, Supplementary Materials) had a strong maximum at around 330 nm. The absence of a shift in the wavelength of the characteristic for C_60_ absorption maximum in DMF [43] suggests the formation of a non-chemical bond between fullerene and PVP. The FA content in the FA-PVP-C_60_ conjugate, assessed by UV-Vis spectroscopy using a molar extinction coefficient of FA in water of 25,220 M^−1^ cm^−1^ at λ_max_ = 280 nm [45], was 8.9 wt.%. The composition of the conjugate (excluding hydrogen) was confirmed by elemental analysis, according to which the C:N:O content was (68.6 ± 2.1) wt.%, (14.5 ± 1.3) wt.%, (16.9 ± 2.0) wt.% and (calc. value C:N:O 70.6 wt.%, 13.7 wt.%, 15.7 wt.%).

It should be noted that the formation of the FA-PVP-C_60_ conjugate is a statistical process. Therefore, only the average number of C_60_ molecules associated with one PVP chain can be estimated in accordance with Equation (1) [46]:

(1) $$n_{C_{60}}=\frac{\left[C_{60}\right]{\cdot M}_{FA\text{-}PVP\text{-}C_{60}}}{\left(1-\left[C_{60}\right]\right)\cdot M_{C_{60}}}\approx 1$$

where M(FA-PVP-C_60_) = 30.5 × 10^3^ g/mol is the molar mass of the conjugate according to capillary viscometry, M(C_60_) = 720 g/mol is the molar mass of fullerene and [C_60_] = 0.028 is the fraction of fullerene in the conjugate.

The average number of FA molecules conjugated to a PVP molecule was assessed similarly. Thus, the macromolecular chain in the conjugate contained, on average, one C_60_ molecule and seven FA molecules. In this case, the final product was most likely a mixture of macromolecules of native PVP and the FA-PVP-C_60_ conjugate. These data are confirmed by the DLS results, which showed fairly wide particle size distributions of the conjugate.

FA is composed of three molecular moieties: pterin (PT), aminobenzoyl (PABA) and glutamate (Glu) (Figure 1B). The UV-Vis spectra of an aqueous solution of the intermediate FA–PVP conjugate and the FA-PVP-C_60_ conjugate (Figure 1C) contain absorption maxima characteristic of FA at 280 nm (peak characteristic of PABA-Glu) and 350 nm (peak characteristic of PT and its derivatives) [47]. As can be noted, the spectrum of FA-PVP-C_60_ does not differ significantly from the spectrum of native FA. When synthesizing FA conjugates, it is important that the PT fragment remains intact to maintain specificity for FR [47].

Possible mechanisms of FA and C_60_ to PVP binding were analyzed using FT-IR spectra of the FA-PVP-C_60_ conjugate, and the data obtained were compared with the spectra of the intermediate FA–PVP conjugate, pure C_60_ and FA (Figure S3, Supplementary Materials). In the FT-IR spectrum of the FA-PVP-C_60_ conjugate (Figure 1D), the bands characteristic of the C_60_ fullerene at 528 cm^−1^, 576 cm^−1^, 1180 cm^−1^, and 1428 cm^−1^ [48] partially overlap with the bands of bond vibrations in PVP (576 cm^−1^). We observed a shift of the strong absorption peak at 1653 cm^−1^, responsible for the C=O stretching vibration in PVP [49] in FA-PVP-C_60_ and FA-PVP. At the same time, the magnitude of this shift is not large, which indicates that no covalent bond was formed between fullerene and PVP. Thus, we can assume that the conjugation of C_60_ with PVP occurred by forming a charge-transfer complex involving the C=O bond of the PVP pyrrolidone ring [50].

The fluorescence emission spectrum of the FA-PVP-C_60_ conjugate labeled with FITC, excited at a wavelength of 350 nm, had a maximum around 445 nm (Figure 1E), which is characteristic of FA in neutral form [45,51]. When the fluorescence excitation wavelength increases to 370 nm, a small shoulder appears in the fluorescence emission spectrum (Figure 1F), whose appearance is associated with the fluorescence of the FITC label, the maximum of which was clearly visible in the emission spectrum at fluorescence excitation of 480 nm (Figure 1G).

Studies have shown that keeping in the dark for 1 h in a temperature range from 25 °C (room temperature (RT)) to 50 °C did not significantly affect the FA-PVP-C_60_ conjugate (Figure S4A, Supplementary Materials). The conjugate was also stable when stored in the dark for 6 months at RT and at 4 °C (Figure S4B,C, Supplementary Materials). The absorbance of conjugate solutions (taking into account a column dilution factor of 2.0) passing through a gel filtration column did not change significantly. Thus, we did not observe the appearance of free FA when the FA-PVP-C_60_ was stored under these conditions. However, when the conjugate was stored at RT under visible light, changes in the UV-Vis spectra were observed. As shown in Figure S4D, there was a hypsochromic shift of the absorption maximum at 280 nm, indicating that the bond between PABA-Glu and PT moieties in folic acid was broken. A similar effect was observed for UV-irradiated FA [47].

**Figure 2:**
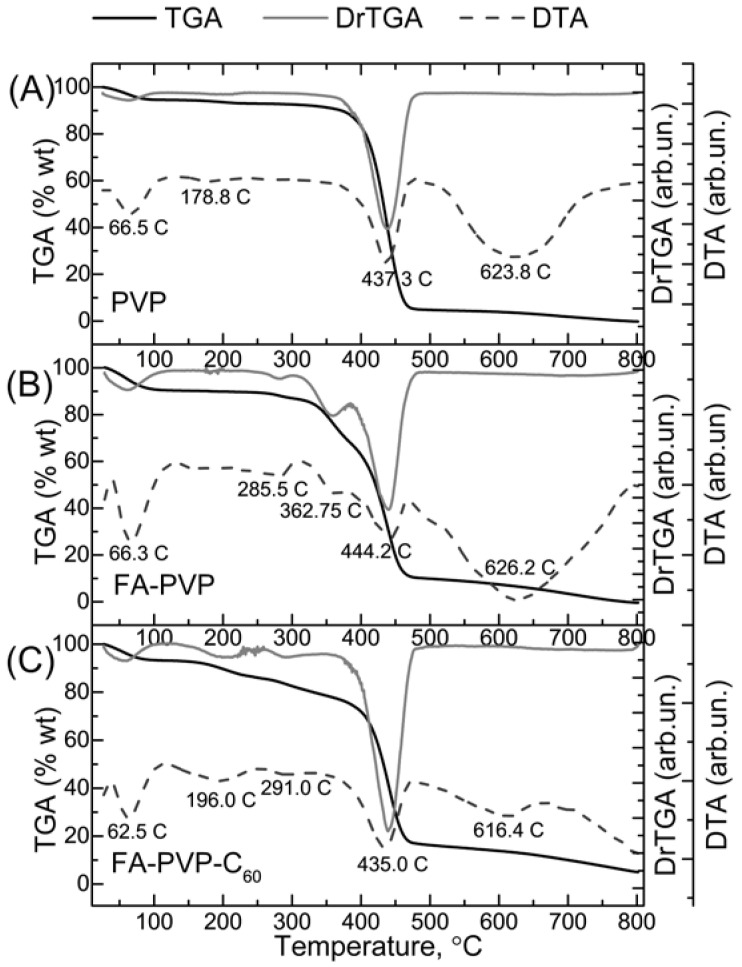
Simultaneous thermal analysis of pure PVP (**A**), intermediate FA–PVP conjugate (**B**), and FA-PVP-C_60_ conjugate (**C**).

To study the thermal behavior of the synthesized conjugates, the decomposition curves of FA-PVP-C_60_ were studied in comparison with the curves of pure PVP and the intermediate FA–PVP conjugate. As can be seen in Figure 2, the behavior of thermal decomposition of FA-PVP-C_60_ differs from the behavior of pure PVP. The main mass loss in the TGA curves of decomposition of the FA-PVP-C_60_ conjugate at 435 °C corresponds to the decomposition of PVP to monomer [52], and this temperature is somewhat lower compared to native PVP. This further indicates the binding of the polymer to C_60_ and FA and was previously observed for other PVP-conjugated nanoparticles [49,53]. The loss of mass in the region of 65–70 °C is associated with the removal of sorbed water. A wide endothermic peak in the DTA curve with a maximum around 70 °C appears due to the occurrence of dehydration reactions. On the DTA curve, the drop in ∆T in the region of 178.8 °C corresponds to the glass transition process, which is not accompanied by a change in enthalpy. The wide endothermic maximum on the DTA curve at 624 °C corresponds to the decomposition of the residues of the lactam group of PVP [54]. The loss of mass in the region of 291 °C is a consequence of the decomposition of FA [55]. The remaining mass at 800 °C present in the conjugate, in contrast to pure PVP and FA-PVP, corresponds to fullerene, which further confirms the presence of C_60_ in the synthesized conjugate.

### 2.2. Particle Size Distributions and ζ-Potential Analysis

The size and stability of nanoparticles in drugs are important characteristics that determine the properties of drugs in vivo. In addition, the activation of receptors located on the cell membrane surface depends on the size of the agents that bind to it [56]. It has been shown that particles up to 300 nm in size are removed from the bloodstream more slowly than larger ones [57]. Particles smaller than 200 nm can be nonspecifically taken up by cells [58]. In this case, the maximum of the enhanced permeability and retention effect (EPR effect) in the tumor is observed [59]. It is also known that the size of fullerene derivatives in aqueous solutions is an important property in terms of their toxicity [60,61] and interaction with biological objects since they tend to form aggregates that can behave differently than individual nanoparticles [62]. Here, targeted delivery of drugs based on fullerene derivatives, their accumulation in the tumor and prevention of the diffusion of nanoparticles back into the vascular bed become of paramount importance [57,63,64].

**Figure 3:**
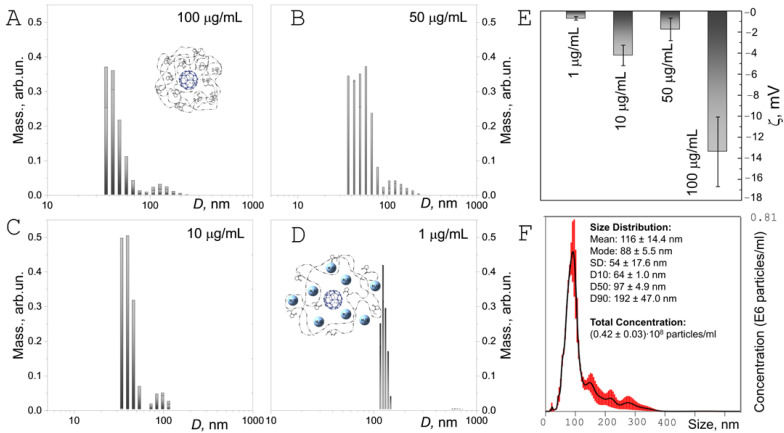
(**A**–**D**) The size distributions of FA-PVP-C_60_ by mass after 24 h incubation in water at 37 °C in the dark, measured by DLS. (**E**) ζ-potential ± SD of FA-PVP-C_60_ in aqueous solution measured by PALS. (**F**) Size distribution (on the inset) and concentration profiles (black curve) of FA-PVP-C_60_ based on NTA. Red error bars indicate ±1 SEM. The insets (**A**,**D**) show a sketch of the FA-PVP-C_60_ conjugate.

We found that FA-PVP-C_60_ conjugates strongly aggregate in an aqueous solution. Similar results were obtained earlier for water-soluble fullerene derivatives with amino acids [65] and polymers [66]. According to dynamic light scattering (DLS) data, at a temperature close to physiological values (37 °C) in aqueous solutions of the conjugate (Figure 3A–C), two dominant groups of aggregates with average sizes of 40 and 150 nm can be distinguished. When the concentration of FA-PVP-C_60_ conjugate in aqueous solution was reduced to 1 μg/mL, only clusters with an average size of 130 nm were observed in the mass distribution of particles (Figure 3D). This effect may be associated with the inclusion of water molecules in a complex with smaller aggregates due to the transfer of electron density from the carbonyl groups of PVP [46], which leads to the formation of looser colloidal structures (shown in the insets to Figure 3A,D). In addition, at a higher concentration of the conjugate, the stabilizing property of PVP is more pronounced, preventing aggregation due to repulsive forces arising from hydrophobic carbon chains interacting with each other. At the same time, at higher concentrations of the conjugate, PVP coils can become more compact due to the attraction of C_60_ fullerenes due to hydrophobic interactions. This is confirmed by ζ-potential measurements, whose absolute values decrease with decreasing conjugate concentration (Figure 3E). According to other sources, the negative value of the ζ-potential of FA-PVP-C_60_ may be associated with the accumulation of PVP oxygen atoms on the C_60_ surface as a result of the chemisorption process [67]. According to the nanoparticle tracking analysis (NTA) data (Figure 3F), the FA-PVP-C_60_ conjugate in aqueous solution formed conjugates with an average size of 116 nm. Considering the reduced sensitivity of NTA to particles smaller than 50 nm, resulting in a shift of the distribution center towards larger sizes [68], these data are consistent with those obtained by DLS.

**Figure 4:**
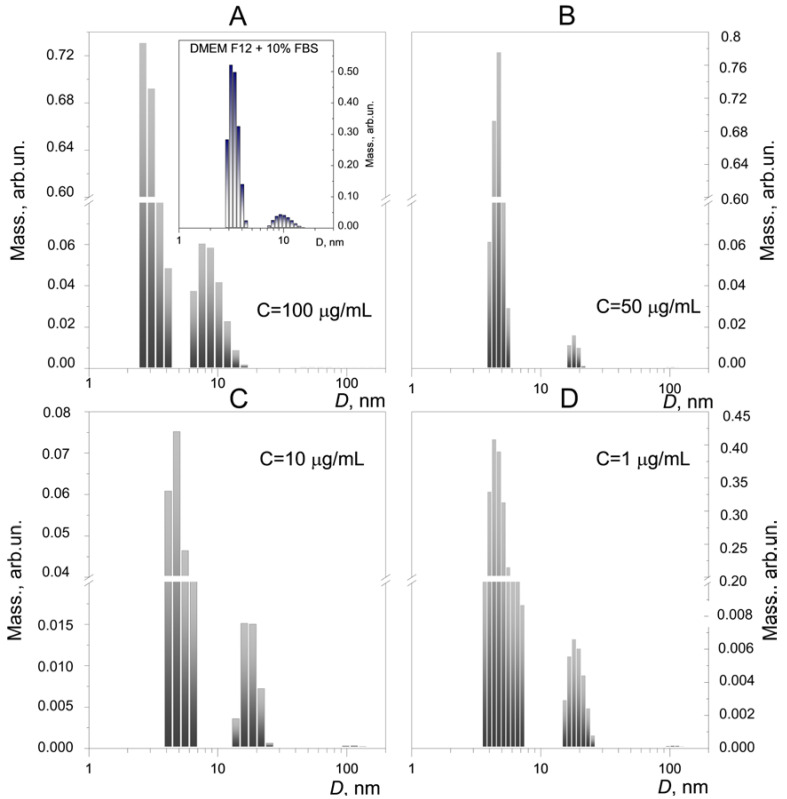
(**A**–**D**) The size distributions of FA-PVP-C_60_ particles by mass after incubation in DMEM F12 + 10% FBS for 24 h at 37 °C in the dark. The inset (**A**) shows the size distributions of pure DMEM F12 + 10% FBS (incubated under the same conditions) particles.

Equally important is the assessment of the behavior of FA-PVP-C_60_ in a cell culture medium, since the particle size matters in their interaction with biological objects and can affect cytotoxicity and the ability to penetrate the cell membrane. In addition, the protein present in the cell culture medium can interact with conjugates, affecting their hydrophobicity, reactivity and ζ-potential [61,69]. Despite the fact that in all samples of the FA-PVP-C_60_ conjugate in DMEM F12 + 10% fetal bovine serum (FBS), as in aqueous solutions, we observed a bimodal particle size distribution (Figure 4), the aggregates in the cell culture medium were significantly smaller. In addition, the particle size of FA-PVP-C_60_ in the cell culture medium was found to be constant over the conjugate concentration range from 1 to 50 μg/mL; the average sizes of smaller and larger aggregates were about 5 and 19 nm, respectively. At the same time, as shown in the insets of Figure 4A, the components of the FBS-containing cell culture medium also formed aggregates with average sizes of 3.5 and 10 nm. Probably, FBS plays the main role in changing the behavior of conjugates in the cell culture compared to aqueous solutions. A similar behavior was also observed for fullerenols; aggregates of these nanoparticles in the FBS-containing cell culture medium were smaller than in a serum-free medium [62]. Unfortunately, we were unable to estimate the zeta potential of conjugate particles in the cell culture medium due to its high electrophoretic mobility and the need for special devices for these measurements [70].

Thus, our data show that the composition of the solvent plays a very important role in the creation of drugs based on fullerenes. In addition, it is necessary to take into account the fact that when the drug is placed in a biological environment, its parameters may change.

### 2.3. Radical Scavenging Activity of FA-PVP-C_60_ Conjugate In Vitro

Problems with using FA as a targeting ligand in targeted drug delivery systems are its low photostability under illumination [71,72] and radiolysis under the influence of ionizing radiation [73]. The presence of fullerene in the FA-PVP-C_60_ conjugate, which is known for its antioxidant properties [74], may be useful in terms of the possible inhibition of photoinduced destruction of FA, as has been shown for other antioxidants [75].

**Figure 5:**
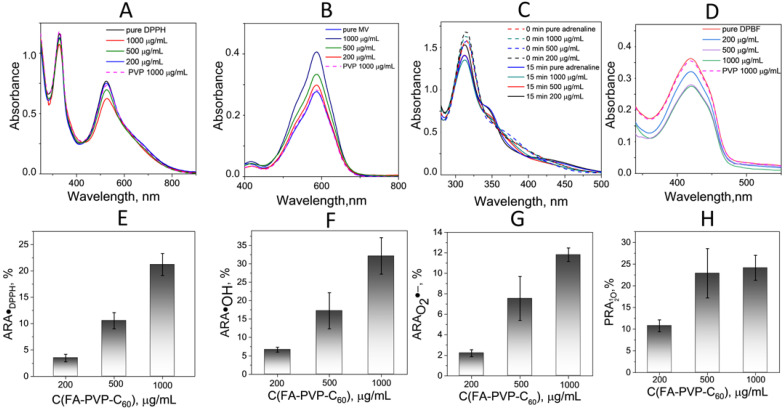
(**A**,**E**) DPPH radical scavenging activity of FA-PVP-C_60_; (**B**,**F**) hydroxyl radical scavenging activity of FA-PVP-C_60_; (**C**,**G**) superoxide radical scavenging activity of FA-PVP-C_60_; (**D**,**H**) singlet oxygen prooxidant activity of FA-PVP-C_60_.

The FA-PVP-C_60_ conjugate demonstrated dose-dependent moderate activity against the model radical 2,2-diphenyl-1-picryl-hydrazyl(˙DPPH) (Figure 5A,B). Also, the FA-PVP-C_60_ conjugate was found to have moderate activity against reactive oxygen species such as hydroxyl radical ^•^OH (Figure 5C,D) and superoxide O_2_^•−^ (Figure 5E,F). At the same time, the pro-oxidant light activity of the FA-PVP-C_60_ conjugate was detected in the oxidation reaction of 1,3-Diphenylisobenzofuran (DPBF) by singlet oxygen ^1^O_2_ generated by irradiation of the photosensitizer Rose Bengal (RB) (Figure 5G,H). In this case, DPBF was found to be oxidized to a greater extent in the presence of the FA-PVP-C_60_ conjugate than in its absence (Figure 5C). At the same time, the pure PVP used for comparison did not have a significant effect on the oxidation state of DPBF.

The detected photodynamic activity of the FA-PVP-C_60_ conjugate is consistent with data obtained previously both for C_60_ fullerene and for its conjugates with polymers [76,77]. At the same time, the fullerene conjugate studied in [66] did not exhibit significant light-induced cytotoxicity.

### 2.4. Toxicity Assay

**Figure 6:**
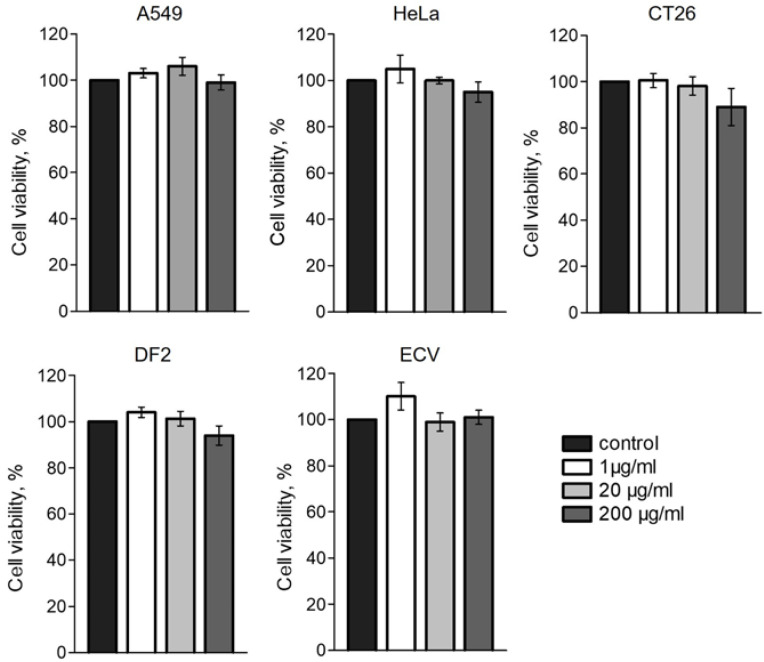
MTT assay. Conjugate FA-PVP-C_60_ was added to the culture 24 h after seeding and was present in the culture medium for 24 h. Control cells do not contain the conjugate. The difference between the control sample and the experimental sample is not significant (*p* ≥ 0.05). *n* ≥ 8 experiments.

The physical characteristics of nanoparticles are closely related to their toxicity, which in turn may depend on the cell type. It was previously shown that C_60_ fullerene itself has low toxicity [78]. However, it can increase with the functionalization of nanoparticles, which is necessary to increase biocompatibility [17]. At the same time, some publications [79,80] assert that C_60_ can be toxic to tumor cells. We analyzed the cytotoxicity of the conjugate containing C_60_ in comparison with the FA–PVP conjugate on several cell lines. We used both healthy (non-transformed) DF2 cells and transformed ECV cells obtained from healthy tissue, as well as transformed HeLa, A549 and CT26 cells, obtained from tumor tissue. Viability was determined using the MTT test, assessing the metabolic activity of cells after 24 h of exposure to the conjugates. During the experiment, the cells were kept mainly in the dark, and manipulations were carried out under dim lighting to eliminate phototoxicity that is likely to occur due to the presence of C_60_ in the conjugate [81], as well as photolysis of FA [47,82]. From the results presented in Figure 6 and Figure S5, it can be concluded that the conjugates at the tested concentrations (1, 20 and 200 μg/mL) did not reduce the viability of all cell lines analyzed when incubated for 24 h. In this case, neither the type of cells nor the level of expression of FR in them mattered. Thus, we have not confirmed the fact that C_60_ is more toxic to tumor cells than to healthy ones, at least as a component of this conjugate.

### 2.5. Conjugate FA-PVP-C_60_ Internalization into Cells

**Figure 7:**
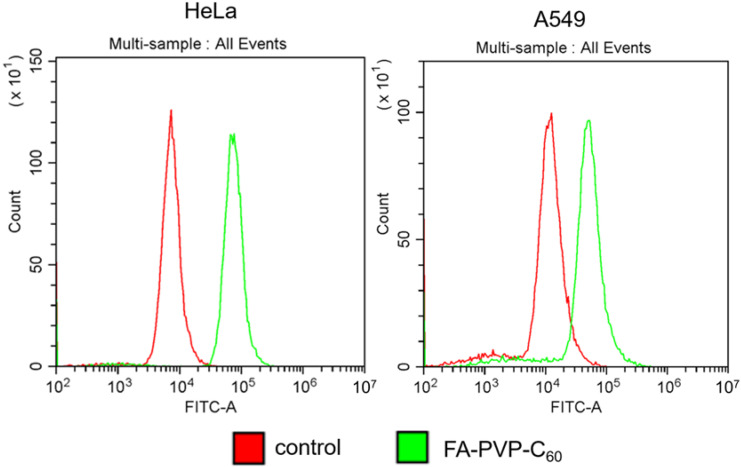
Analysis of FA-PVP-C_60_ conjugate internalization into cells with different folate receptor expression levels. Flow cytometry data.

The targets of action for most antitumor drugs are located inside the cell, hence the effectiveness of drug internalization as part of a targeted delivery system determines the result of its action. It is assumed that the uptake of the conjugate with FA occurs due to FR-mediated endocytosis [10,39]. For analysis, we used tumor cells with different expressions of FR on the surface. According to data in the literature, HeLa cells are characterized by overexpression, while A549 cells are characterized by low expression of FR [39,83,84,85,86]. The penetration efficiency of the FITC-labeled conjugate was assessed by the fluorescence intensity of the cells using a flow cytometer as well as a confocal microscope at a wavelength of 488 nm. According to cytometry data, the fluorescence intensity in HeLa cells compared to the control is higher than in A549 cells (Figure 7). Consequently, the conjugate enters cells with a higher level of expression of FR more efficiently.

**Figure 8:**
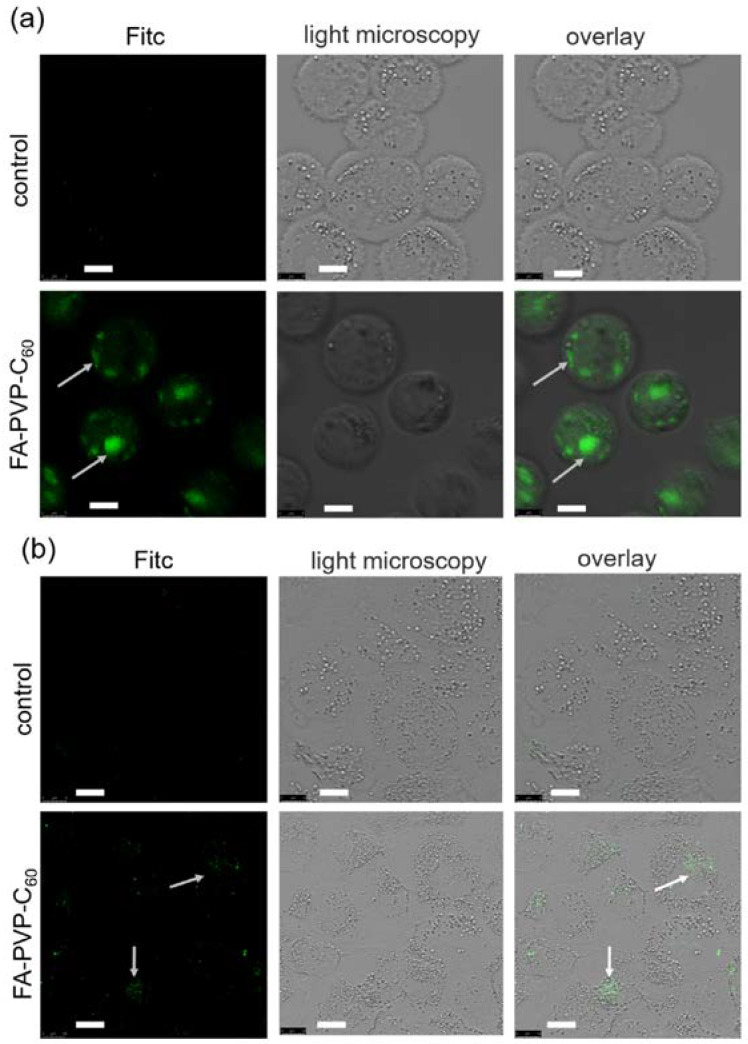
Confocal luminescence microscopy of the cells incubated with FITC-labeled FA-PVP-C_60_ (**a**) Hela cells, (**b**) A549 cells. Arrows show fluorescence signals. Scale bar, 8 μm.

Confocal images confirm this conclusion (Figure 8a,b). Intracellular fluorescence is present in both cell lines; however, in HeLa cells with the overexpression of FR, higher emission is observed compared to the A549 cell line. This means that the conjugate accumulates in them in greater quantities. It can be noted that the conjugate is unevenly distributed inside the cell, thus in the future, it will be interesting to study its colocalization with nuclear material and cytoplasmic organelles, which is necessary when designing targeted effects on functions, that is, increasing the effectiveness of drugs [57].

Similar drugs created by other researchers showed similar behavior. Thus, in [87], using a nucleophilic addition reaction, conjugates of C_60_ fullerene with FA were obtained for use in photodynamic therapy. It was shown that the uptake of conjugates by FR-positive HeLa cells is significantly higher than by FR-negative N2a cells. J. Fan et al. [39] found that after 2 h of incubation with DOX-hydrazone-fullerenol-FA nanoparticles, FR-positive HeLa cells showed much brighter fluorescence than FR-negative cells (L929 and A549). Our data confirm the fact that fullerene conjugates with folic acid do not exhibit obvious nonspecific cellular uptake but have selectivity towards cells with increased expression of folate receptors, being transported into them through an FR-mediated endocytic process.

### 2.6. Cell Cycle Analysis

**Figure 9:**
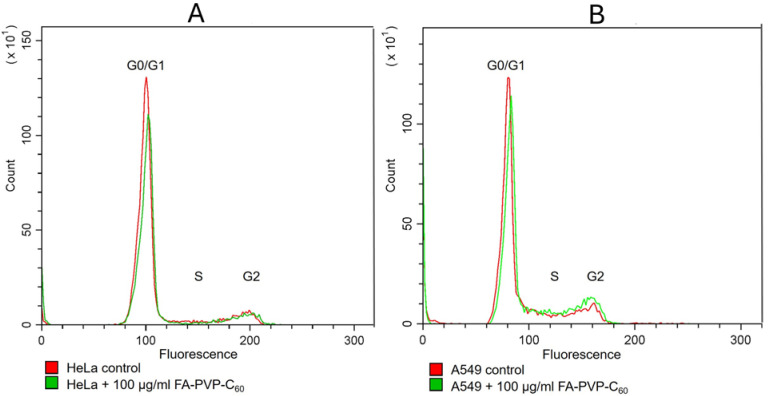
Effect of the FA-PVP-C_60_ conjugate on the cell cycle in lines with different expressions of FR. (**A**) HeLa—FR-positive cells, (**B**) A549—FR-negative cells. G0, G1, G2, S—phase of the cell cycle preceding mitosis (G0—resting phase, G1—growth phase, S—phase of DNA replication, G2—period of protein synthesis and preparation of the cell for mitosis).

A higher degree of internalization of the conjugate into FR-positive HeLa cells did not correlate with increased toxicity, as demonstrated by the results of the MTT test. However, there is a possibility that a drug that does not affect the dehydrogenase activity of mitochondria may affect the cell cycle [88]. Using flow cytometry, we analyzed the cell cycle of the HeLa and A549 lines and did not see a significant difference either between the control and experimental cells in either line or between these lines, which indicates that the conjugate does not have pronounced cytostatic properties. The analysis data are presented in Figure 9 and Table 1.

## 3. Materials and Methods

### 3.1. FA-PVP-C_60_ Conjugate Synthesis and Characterization

Fullerene C_60_ with a purity of 99.9+ wt.% was synthesized at the Petersburg Nuclear Physics Institute named by B.P. Konstantinov of the National Research Center “Kurchatov Institute” [89]. Its purity was monitored using high-performance liquid chromatography (HPLC) on an LC-20 Prominence liquid chromatograph (Shimadzu, Kyoto, Japan), IR-Fourier spectrometry in the 4000–400 cm^−1^ range on an IRTracer-100 spectrometer with an attenuated total reflectance (ATR) attachment (Shimadzu, Kyoto, Japan) and UV-Vis spectrometry on an SF-2000 spectrophotometer (OKB Spectr LLC, Saint-Petersburg, Russia). The crystal structure of the sample was previously confirmed using powder X-ray diffractometry [90].

To synthesize the conjugate, 100 mg FA (purity 98 wt.%, Sisco, Mumbai, India) and 1 g PVP K25 (average molecular weight 30 kDa, Shanghai Yuking Water Soluble Material Tech Co., Ltd., Shanghai, China) were mixed in 50 mL N,N-Dimethylformamide (DMF, Vecton JSC, Saint-Petersburg, Russia) for 2 h at a temperature of 40 °C. Then, a 150 μg/mL solution of C_60_ in DMF was added to the FA–PVP solution and stirred at 60 °C for 24 h. The resulting conjugate was centrifuged, evaporated, and vacuum-dried to remove solvent impurities. The solubility of the conjugate in water was ~50 mg/mL. For the subsequent analysis of the conjugate internalization into cells, FA-PVP-C_60_ was labeled with fluorescein isothiocyanate (FITC). To do this, 100 μL of a solution of FITC (Thermo Fisher Scientific, Waltham, MA, USA) in dimethyl sulfoxide (DMSO, purity 99.5%, Vecton JSC, Saint Petersburg) with a concentration of 1 mg/mL was added to 5 mL of a conjugate solution with a concentration of 5 mg/mL in water. The mixture was sonicated at 40 kHz for 15 min and kept in the dark at 4 °C for 8 h to allow the reaction to occur, which was then quenched by blocking the remaining unreacted isothiocyanate groups by adding ammonium chloride (Vecton JSC, Saint-Petersburg, Russia) to the final concentration of 50 mM. Next, the conjugate was purified from free FA and unreacted FITC by gel filtration on Sephadex G-25 (HiTrap Desalting, Cytiva, Marlborough, MA, USA) using deionized water as an eluent.

The solid-state ^13^C NMR studies were carried out on a Bruker Avance III 400WB spectrometer (Bruker Corporation, Billerica, MA, USA). A two-channel sensor equipped with a magic angle sample rotation (MAS) system was used. The sample was placed in a 4 mm zirconium oxide rotor and rotated at a frequency of 12.5 kHz. Tetramethylsilane ((TMS), Sigma Aldrich, St. Louis, MO, USA) was used as an external standard. For excitation, a cross-polarization sequence (CP/MAS) was used with a 2 s relaxation delay time and 2 ms and 8 ms contact times; a sequence of direct excitation with decoupling from protons with a 30 s relaxation delay time and 3.2 μs of exciting pulse duration. Elemental analysis of the conjugate was performed using SEM Tescan Vega 3 SBH (Tescan, Brno, Czech Republic) using the AdvancedAztecEnergy elemental composition determination system based on an X-act semiconductor energy-dispersive detector (Oxford Instruments, Abingdon, UK). To record IR spectra in the range of 4000–400 cm^−1^, an IRTracer-100 infrared Fourier spectrometer (Shimadzu, Kyoto, Japan) with an ATR attachment was used. Absorption spectra were recorded by means of an SF-2000 spectrophotometer (OKB Spectr LLC, Saint Petersburg, Russia). Fluorescence excitation and emission spectra were recorded by means of a CM2203 spectrofluorometer (Solar CJSC, Minsk, Republic of Belarus). Thermal analysis was carried out using a TG/DTA simultaneous measuring instrument DTG-60 (Shimadzu, Kyoto, Japan) in a temperature range from 26 to 800 °C at a heating rate of 10 °C/min in a nitrogen atmosphere at a 200 mL/min gas flow rate. A capillary viscometry was used to determine the FA-PVP-C_60_ conjugate and pure PVP molar masses.

### 3.2. Particle Size Distribution and Zeta Potential Measurements

Experiments to determine particle sizes in solutions of the FA-PVP-C_60_ conjugate with a concentration of 1100 μg/mL were carried out after 24 h of incubation under standard conditions (37 °C, 5% CO_2_) in deionized water or DMEM F12 cell culture medium with glutamine (Biolot, Saint-Petersburg, Russia), antibiotics (penicillin and streptomycin (Biolot, Saint-Petersburg, Russia)) and 10% FBS (Biolot, Saint-Petersburg, Russia)) by dynamic light scattering (DLS). The studies were performed using the dynamic light-scattering method on a Photocor Compact-Z analyzer (Photocor LLC, Moscow, Russia) with a laser wavelength of 654 nm and a maximum light beam power of 25 mW, at a scattering angle of 90° and a stabilized sample temperature of 37.0 °C. Size distributions of light-scattering particles based on contribution to light scattering and the masses of particles were obtained by analyzing the autocorrelation function of the intensity of light scattered by the samples using DynaLS software (Vers. 2.9.1, Dr. Alexander Goldin, Alango Ltd., Tirat Carmel, Israel). Measurements of the ζ-potential using laser Doppler anemometry were carried out using the same device. Analysis of the Doppler shift of the studied samples was carried out using the PALS (Phase-Analysis Light Scattering) method. The stability of the determined size distribution of particles and the ζ-potential were determined by at least three measurements of each sample. In addition, the size and concentration distributions of FA-PVP-C_60_ aqueous solutions were measured using nanoparticle tracking analysis (NTA) on a NanoSight LM10 (Malvern, Worcestershire, UK). The FA-PVP-C_60_ solution was diluted to obtain the concentration in the operating range of the device (~10^8^ particles/mL).

### 3.3. Radical Scavenging Activity of FA-PVP-C_60_ Conjugate In Vitro

#### 3.3.1. DPPH Scavenging Activity

The antiradical activity of FA-PVP-C_60_ with the stable radical DPPH was studied using UV-Vis spectrometry on an SF-2000 spectrophotometer (OKB Spectr LLC, Saint-Petersburg, Russia). For this purpose, a solution of DPPH (extra pure, 95%, Sisco, Mumbai, India) in ethanol with a concentration of 130 μM and aqueous FA-PVP-C_60_ solutions at concentrations of 200–1000 μg/mL were prepared. After incubation in a 37 °C water bath for at least 1 h, equal volumes of DPPH and FA-PVP-C_60_ solutions were placed in a quartz cuvette, and absorbance was measured at 525 nm. A mixture of equal volumes of ethanol and a water solution of FA-PVP-C_60_ of the appropriate concentration was used as a blank sample. The solutions were quickly mixed, the quartz cuvettes were covered with a lid to prevent ethanol evaporation and measurements were taken immediately at room temperature in the dark. The experiment was independently repeated three times.

The antiradical activity of FA-PVP-C_60_ *ARA_DPPH·_* was determined at 60 min after the start of the ^•^DPPH reduction reaction using Equation (2):

(2) $${ARA}_{DPPH\cdot }=\frac{\left(A_{DPPH\cdot }-A_{sample}\right)}{A_{DPPH\cdot }}\cdot 100\%$$

where${\;A}_{sample}$ and $A_{DPPH}$ are absorbance at 525 nm of ^•^DPPH solutions in the presence and absence of FA-PVP-C_60_, respectively.

#### 3.3.2. Hydroxyl Radicals Scavenging Activity

The ability of FA-PVP-C_60_ to scavenge hydroxyl radicals was studied using spectrophotometric determination of the destruction degree of methyl violet (MV) when it reacts with ·OH [91]. The hydroxyl radicals were generated during the Fenton reaction Fe^2+^/Fe^3+^, occurring in the presence of H_2_O_2_. A mixture of equal volumes of aqueous solutions of 30 μM MV (LenReactiv, Saint-Petersburg, Russia), 50 mM H_2_O_2_ (Vecton JSC, Saint-Petersburg, Russia) and 0.4 mM iron (II) sulfate 7-hydrate (Vecton JSC, Saint-Petersburg, Russia) in deionized water, and an aqueous solution of FA-PVP-C_60_ at concentrations of 200–1000 μg/mL were incubated in a water bath at 37 °C for 60 min. A similar mixture was used as a blank experiment, replacing the MV solution with deionized water. The absorbance of the samples was measured at 585 nm using an SF-2000 spectrophotometer (OKB Spectr LLC, Saint-Petersburg, Russia). The *A*_•OH_ absorbance was measured by replacing the FA-PVP-C_60_ solution with an equal volume of deionized water. The experiments were repeated independently three times.

The scavenging activity (%) of hydroxyl radical *ARA*_•OH_ was calculated using the following Equation (3):

(3) $${ARA}_{•OH}=\frac{\left(A_{•OH}-A_{sample}\right)}{A_{•OH}}\cdot 100\%$$

where $A_{•OH}\;and\;A_{sample}\;$are absorption at 585 nm 60 min after the start of the reaction of the mixture of solutions of MV, H_2_O_2_ and iron (II) sulfate 7-hydrate in the absence and presence of FA-PVP-C_60_, respectively.

#### 3.3.3. Superoxide Radicals Scavenging Activity

The ability of FA-PVP-C_60_ to remove superoxide radicals O_2_^•−^ was determined by the degree of inhibition of the adrenaline autoxidation reaction. A detailed description of the experiment was presented previously [30]. The experiment was independently repeated three times.

The scavenging activity (%) of superoxide radical anion ${ARA}_{O_{2˙}^{-}}$ was calculated using the following Equation (4):

(4) $${ARA}_{O_{2˙}^{-}}=\frac{\left(A_{O_{2˙}^{-}}-A_{sample}\right)}{A_{O_{2˙}^{-}}}\cdot 100\%$$

where $A_{O_{2˙}^{-}}\;and\;A_{sample}\;$are absorption 15 min after the start of the reaction of solutions in carbonate buffer (pH 10.7) of adrenaline hydrochloride (Moscow Endocrine Plant, Moscow, Russia) and a similar solution in the presence of FA-PVP-C_60_ at concentrations of 200–1000 μg/mL, respectively.

#### 3.3.4. Singlet Oxygen Pro-oxidant Activity

Singlet oxygen ^1^O_2_ was generated by the RB photosensitization reaction (RB, Sisco, Mumbai, India). The ability of FA-PVP-C_60_ to generate ^1^O_2_ was determined by the degree of its influence on the bleaching of DPBF (Leap Chem, Hong Kong, China), which is oxidized by ^1^O_2_ [92]. A 1 mL volume of 0.1 mM of RB in water, 100 μL of a 1 mM solution of DPBF in DMSO and 1 mL of an aqueous solution of FA-PVP-C_60_ at concentrations of 200–1000 μg/mL were placed in a cuvette. To generate ^1^O_2_, the cuvette was irradiated with a laser (λ = 532 nm) with a power of 1 mW (Yiwu Zhangkun Electronic Commerce Co., Ltd., Jinhua, Zhejiang, China) for 8 min. The oxidation of DPBF was monitored spectrophotometrically on an SF-2000 (OKB Spectr LLC, Saint-Petersburg, Russia) by reducing the absorption maximum to a wavelength of 418 nm relative to a similar mixture and replacing DPBF with an equal volume of pure DMSO. The experiments were independently repeated three times.

The generation activity (%) of singlet oxygen PRAO21 was calculated using the following Equation (5):

(5) PRAO21=Asample−AO21AO21·100%

where AO21 and Asample are absorption 8 min after the start of the oxidation reaction of DPBF and a similar solution in the presence of FA-PVP-C_60_ at concentrations of 200–1000 μg/mL, respectively.

### 3.4. Cell Cultures

The following lines were used in the investigation: DF2, human dermal fibroblasts; ECV, human umbilical vein endothelial cells; A549, human lung cancer cells (with a low level of FR expression), as well as HeLa, human cervical carcinoma cells; and CT26, mouse colorectal adenocarcinoma (with a high level of FR expression). DF2 cells were obtained from the shared Vertebrate Cell Culture Collection research facility, while the rest were obtained from the collection at the Petersburg Nuclear Physics Institute named by B.P. Konstantinov of the National Research Center “Kurchatov Institute”. Cells were cultured under standard conditions (37 °C, 5% CO_2_) in DMEM F12 medium with glutamine (Biolot, Saint-Petersburg, Russia), with the addition of antibiotics (penicillin and streptomycin (Biolot, Saint-Petersburg, Russia)) and 10% FBS (Biolot, Saint-Petersburg, Russia). When cultivating DF2, fibroblast growth factor was added to the medium at a concentration of 20 ng/mL (cat#PSG060-10. Lot#16F0519F2, Sci-Store, Skolkovo, Russia).

### 3.5. Toxicity Assay

The toxicity of the FA-PVP-C_60_ conjugate was analyzed using the MTT assay. The ECV, A549, CT26 and HeLa cells were seeded in a 96-well plate, and after 24 h of incubation, the medium was replaced with fresh medium containing antibiotics, 10% FBS and the conjugate (FA-PVP-C_60_) at final concentrations of 1, 20 and 200 μg/mL. After another 24 h, tests were conducted as described in [93]. DF2 cells were grown until they reached 75% confluency; otherwise, the procedure was similar to that described above. Intact cells without the addition of the conjugate were always used as a control, with the medium replaced at the same time intervals as in the experiment. The absorbance of each well was measured using a Multiskan FC spectrophotometer (Thermo Fisher Scientific, Waltham, MA, USA) at a wavelength of 540 nm. The obtained data were processed using the KyPlot 6.0 program (KyensLab Inc., Tokyo, Japan), and Dunnett’s test was conducted. Statistical significance was considered at *p* < 0.05.

To analyze the effect of FA-PVP-C_60_ on the cell cycle, cells were grown in 6-well plates for 24 h. Then, the conjugate was added at a concentration of 10 or 100 μg/mL for 24 h. After incubation with the conjugate, the cells were washed with Versene solution (Biolot, Saint-Petersburg, Russia), removed with Trypsin-Versene solution (Biolot, Saint-Petersburg, Russia), centrifuged for 5 min at 1000 rpm and the supernatant removed. Then, 0.1% Triton in phosphate-buffered saline (Biolot, Saint Petersburg, Russia) and 20 μg/mL propidium iodide (Sigma Aldrich, St. Louis, MO, USA) were added to the cells. The cells were resuspended, and after 5 min, the cell cycle was analyzed on a CytoFLEX B3-R2-V2 flow cytometer (Beckman Coulter, Brea, CA, USA) (20 thousand cells per experiment).

### 3.6. Internalization into Cells Assay

Conjugate internalization data were obtained on a CytoFLEX B3-R2-V2 flow cytometer (Beckman Coulter, Brea, CA, USA). The A549 and Hela cells were grown in 6-well plates. After 24 h, FITC-labeled conjugates were added at a concentration of 100 μg/mL. After 3 h of incubation, control (without conjugate) and experimental cells were washed with the culture medium, then with Versene solution, disaggregated with Trypsin-Versene solution and centrifuged for 5 min at 1000 rpm. A 300 μL volume of fresh medium was added to the sediment and mixed. At least 20 thousand cells per sample were analyzed. Photographs of cells were obtained for the same samples. Cells were placed on a glass slide, covered with a coverslip and analyzed using a Leica TCS SP5 SMD FLCS laser scanning confocal microscope (Leica Microsystems, Wetzlar, Germany) at 40× magnification with oil immersion.

### 3.7. FA Stability

The stability of the FA in conjugate under changes of temperature was studied by keeping the aqueous solution of FA-PVP-C_60_ in the dark in a water bath for 1 h at temperatures of 25 °C (room temperature, (RT)), 37 °C, 45° C and 50 °C. After this, absorbance was measured. The stability of FA in the conjugate aqueous solution over storage for 6 months at RT and 4 °C was analyzed by measuring the absorption of the solution every 2 months. The release of FA from the conjugate was assessed by the absorbance change (taking into account the dilution factor) of the solution passed through the Sephadex G-25 desalting column (HiTrap Desalting, Cytiva, Marlborough, MA, USA) compared to the initial solution. The absorbance of the conjugate aqueous solution, stored for 6 months at RT under visible light, was also measured every two months. Absorbance in all experiments, independently repeated three times, was measured using a spectrophotometer SF-2000 (OKB Spectr LLC, Saint-Petersburg, Russia).

## 4. Conclusions

The synthesized FA-PVP-C_60_ conjugate has the parameters necessary for use in therapy or diagnostics. Namely, FA-PVP-C_60_ had good biocompatibility; the particle size of the conjugate both in aqueous solutions and in cell culture media containing salts, amino acids, proteins and other components of biological fluids allowed it to be internalized into tumor cells. In addition, the increased accumulation of the conjugate in cells with overexpression of folate receptors indicates the possibility of using it for delivery to target cells. At the same time, the accumulation of the drug does not lead to a decrease in the viability of FR-positive cells compared to FR-negative ones, since the conjugate demonstrates the absence of toxicity for the different types of cells used in our study. However, this study has limitations: we did not evaluate conjugate internalization into normal cells and the aggregation stability of FA-PVP-C_60_ solutions during storage. Despite the fact that our data contradict previously obtained information about the increased toxicity of C_60_ towards tumor cells, this fact opens up a good opportunity for further modification of the obtained conjugate as a means for photodynamic therapy or as a targeted radiopharmaceutical. We are currently developing this type of radiopharmaceutical by encapsulating a radioactive metal in a fullerene molecule and studying it in vitro and in vivo as a universal platform for tumor theranostics.

## Figures and Tables

**Table 1:** Distribution of cells according to phases of the cell cycle after 24 h of incubation in a cell culture medium containing the FA-PVP-C_60_ conjugate.

Cell Cycle Phases	G0/G1	S	G2
	% cells		
HeLa control	73.58%	6.94%	14.21%
HeLa + FA-PVP-C_60_	69.22%	9.69%	15.27%
A549 control	66.88%	12.07%	14.25%
A549 + FA-PVP-C_60_	62.70%	14.25%	16.89%

## Data Availability

Data are contained within the article or Supplementary Materials.

## Supplementary Materials

The following supporting information can be downloaded at: https://www.mdpi.com/article/10.3390/ijms25105350/s1.
