# Supplementary material for: Fullerene C60 Conjugate with Folic Acid and Polyvinylpyrrolidone for Targeted Delivery to Tumor Cells
Source: Int J Mol Sci. 2024 May 14;25(10):5350. doi: 10.3390/ijms25105350 (PMC11120752; doi:10.3390/ijms25105350)
Supplement: Supplementary file 1 [file ijms-25-05350-s001.zip › ijms-2994290-supplementary.pdf]

Supplementary Materials.

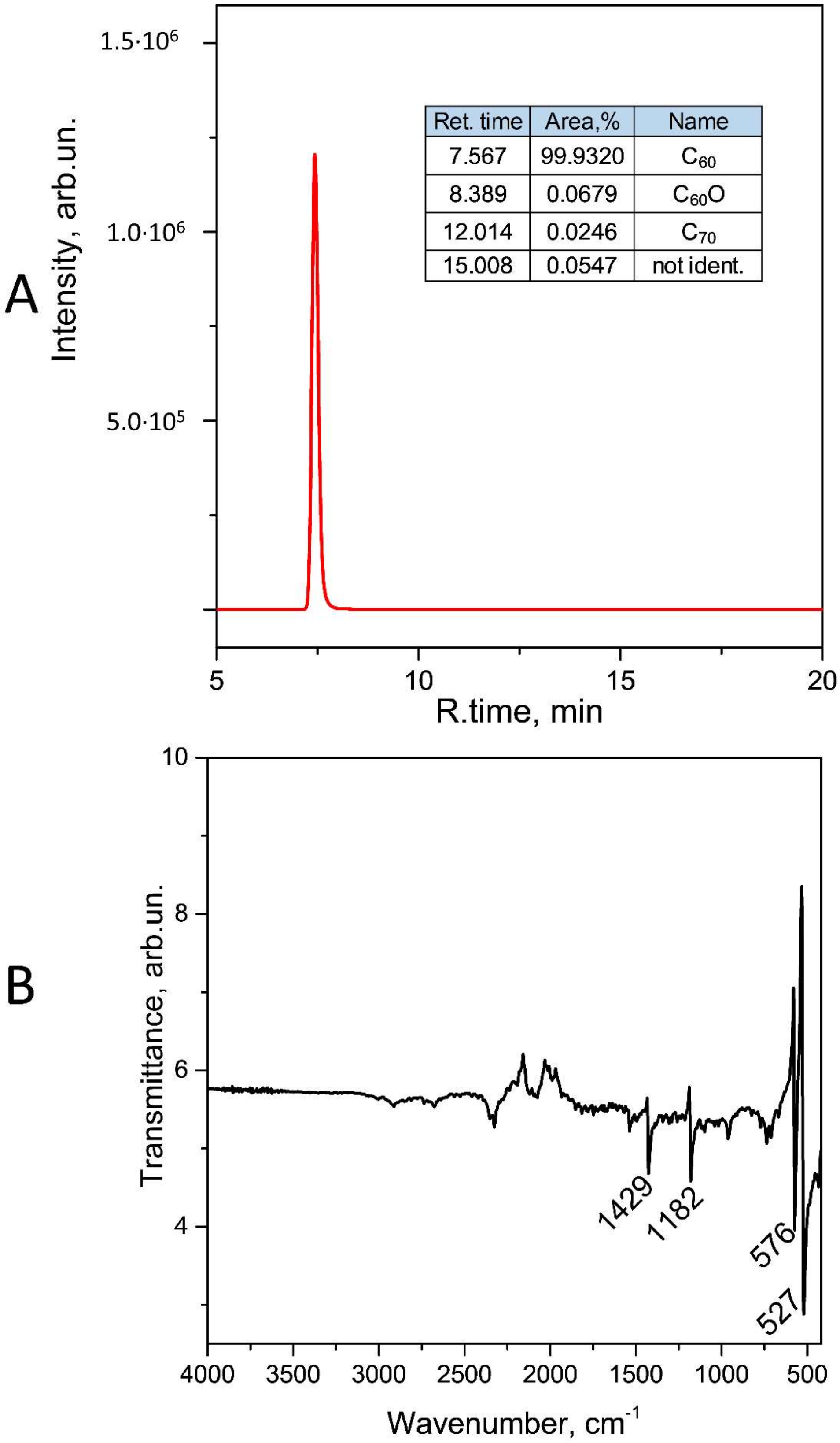

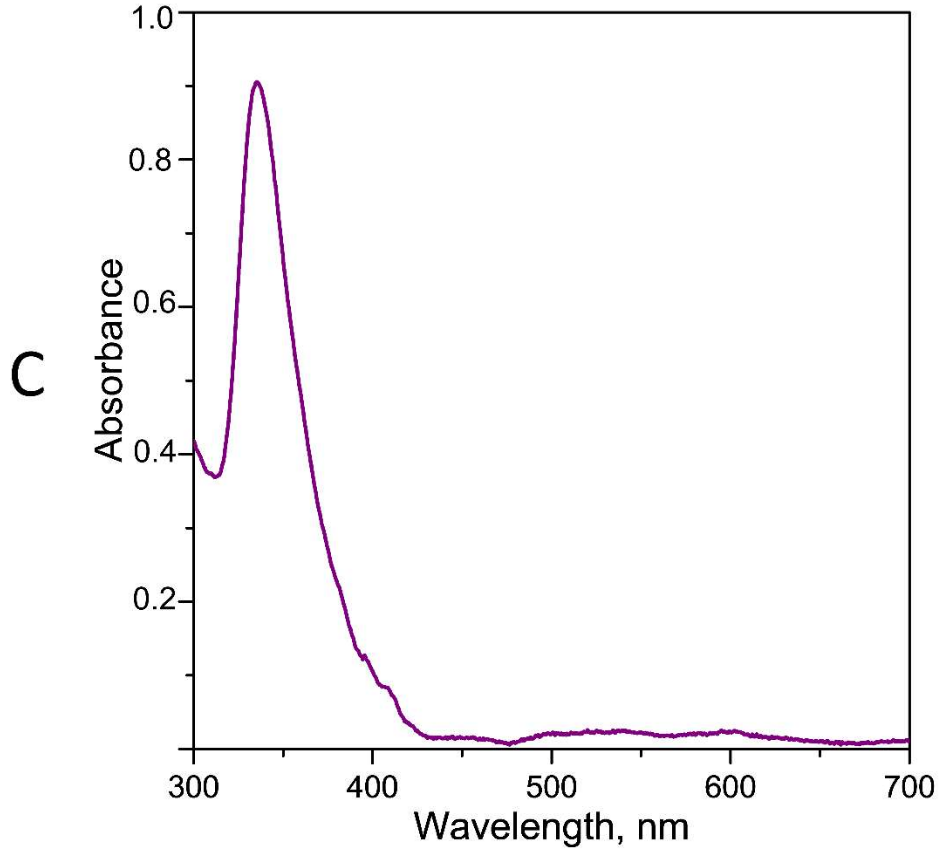

Figure S1. Characterization of pure C_{60}. A – Chromatogram of C_{60} in o-xylene, eluent-toluene, flow rate 1 ml/min, COSMOSYL Buckyprep 4.6 mm I.D. x 250 mm column (Nacalai tsq., Kyoto, Japan);B – FT-IR-spectrum of C_{60}; C – UV/Vis- spectrum of C_{60} in o-xylene

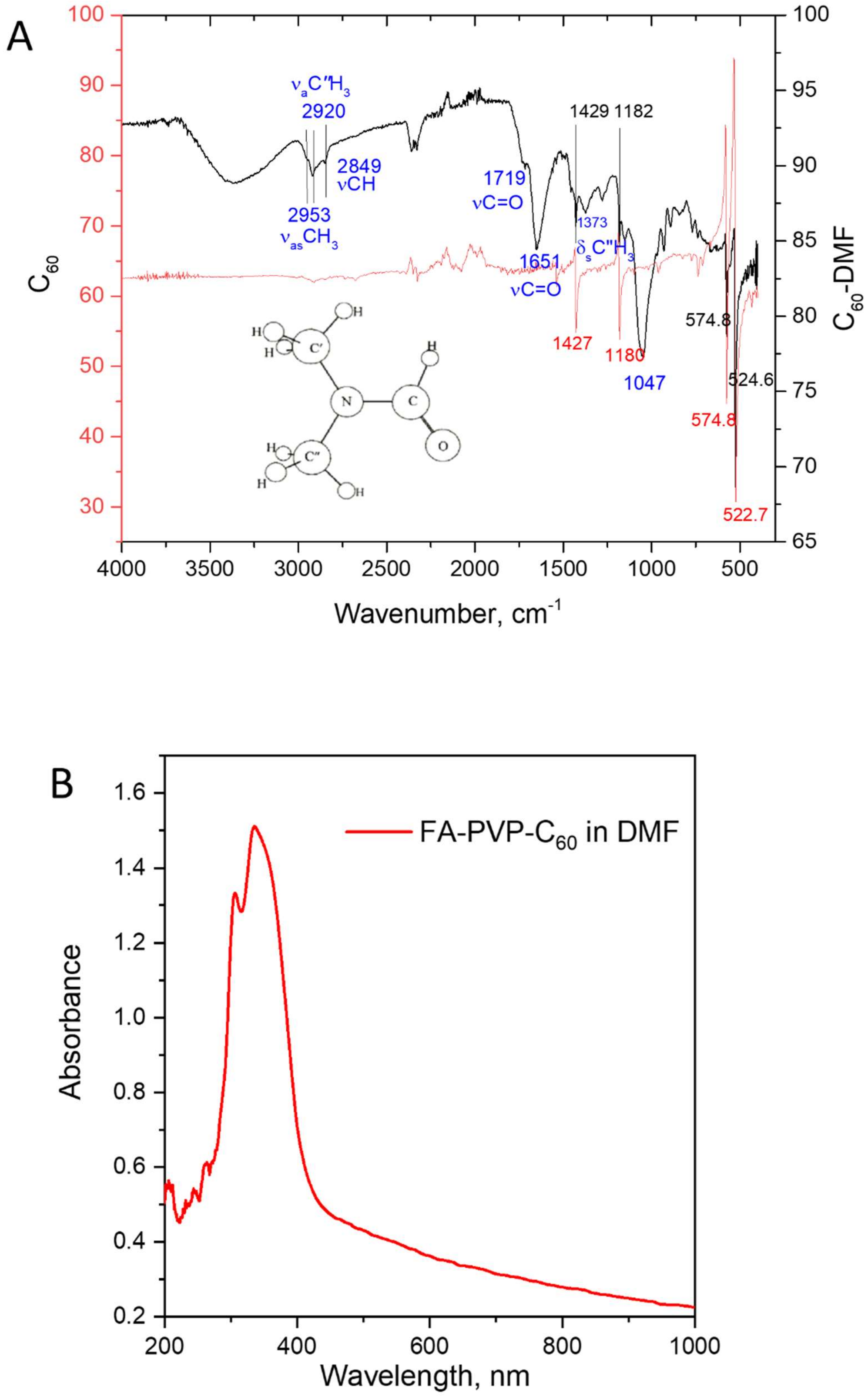

Figure S2. A – FT-IR-spectrum of C_{60}-DMF extract in comparison with the spectrum of pure C_{60}; B – UV-Vis- spectrum of FA-PVP-C_{60} in DMF

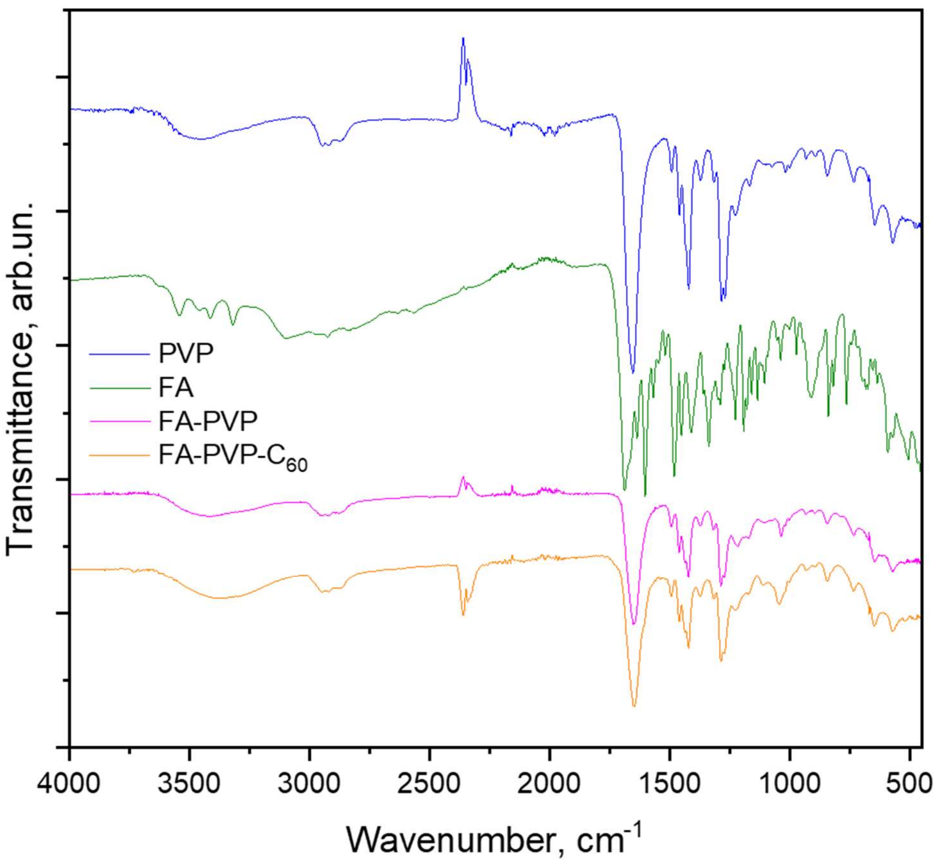

Figure S3. FT-IR-spectra of pure PVP, FA, FA-PVP and FA-PVP-C_{60} conjugates

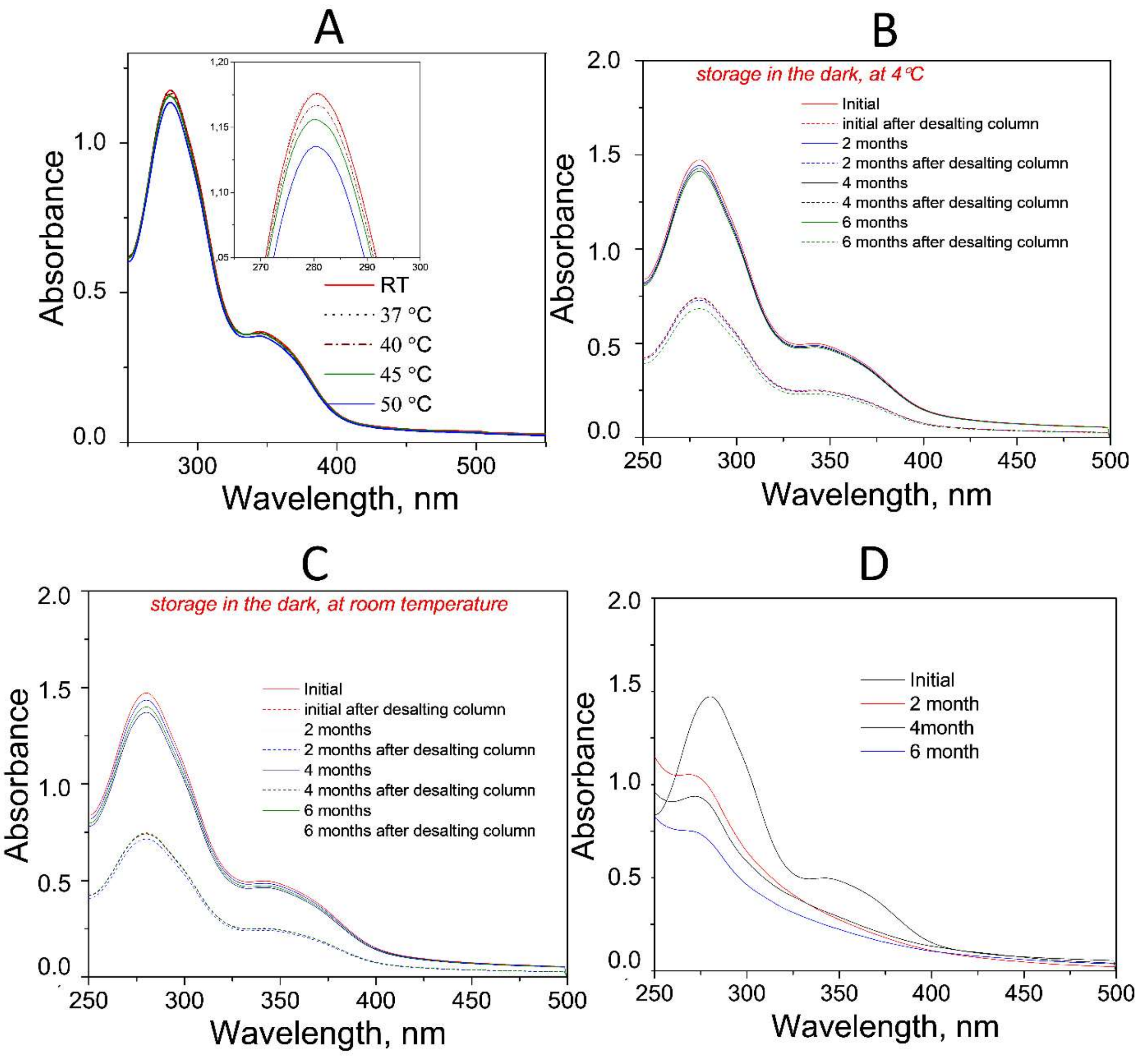

Figure S4. FA stability in conjugate. **(A)** UV-Vis spectra of FA-PVP-C_{60} kept in a thermostat for a 1 h at a temperature from 25 °C (RT) to 50 °C. UV-Vis spectra of FA-PVP-C_{60} stored in the dark at 4°C **(B)** and RT **(C).** (**D**) UV-Vis spectra of FA-PVP-C_{60} stored at RT under the visible light

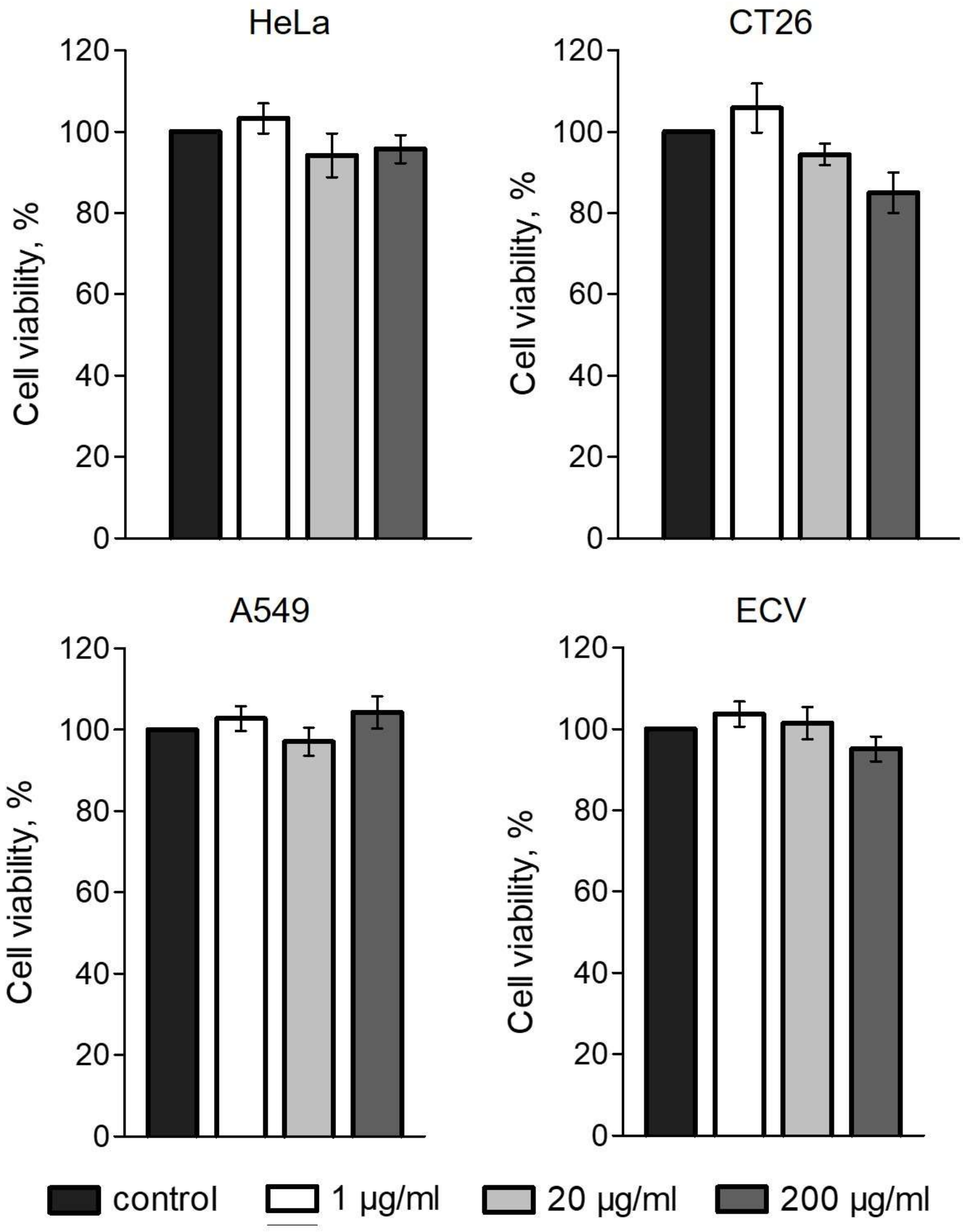

Figure S5. Conjugate FA-PVP MTT assay. FA-PVP were added to the culture 24 h after seeding and were present in the culture medium for 24 h. Control cells without FA-PVP. The difference between the control sample and experimental sample is unreliable (P ≥ 0.05). $n \geq 8$ experiments
